# Association between dietary acid load and the risk of cardiovascular disease: nationwide surveys (KNHANES 2008–2011)

**DOI:** 10.1186/s12933-016-0436-z

**Published:** 2016-08-26

**Authors:** Eugene Han, Gyuri Kim, Namki Hong, Yong-ho Lee, Dong Woo Kim, Hyun Joon Shin, Byung-Wan Lee, Eun Seok Kang, In-Kyu Lee, Bong-Soo Cha

**Affiliations:** 1 Division of Endocrinology, Department of Internal Medicine, Yonsei University College of Medicine, Seoul, Korea; 2 Graduate School, Yonsei University College of Medicine, Seoul, Korea; 3 Institute of Endocrine Research, Yonsei University College of Medicine, Seoul, Korea; 4 Department of Home Economics, Food and Nutrition, Korea National Open University, Seoul, Korea; 5 Department of Nutrition, Harvard School of Public Health, Boston, MA USA; 6 Department of Medicine, Baylor University Medical Center and Baylor Jack and Jane Hamilton Heart and Vascular Hospital, Dallas, TX USA; 7 Division of Endocrinology, Department of Internal Medicine, Kyungpook National University School of Medicine, Daegu, Korea

**Keywords:** Diet, Atherosclerosis, Risk factors, Epidemiology

## Abstract

**Background:**

Acid–base imbalance has been reported to increase incidence of hypertension and diabetes. However, the association between diet-induced acid load and cardiovascular disease (CVD) risk in the general population has not been fully investigated.

**Methods:**

This was a population-based, retrospectively registered cross-sectional study using nationally representative samples of 11,601 subjects from the Korea National Health and Nutrition Examination Survey 2008–2011. Individual CVD risk was evaluated using atherosclerotic cardiovascular disease (ASCVD) risk equations according to 2013 ACC/AHA guideline assessment in subjects aged 40–79 without prior CVD. Acid–base status was assessed with both the potential renal acid load (PRAL) and the dietary acid load (DAL) scores derived from nutrient intake.

**Results:**

Individuals in the highest PRAL tertile had a significant increase in 10 year ASCVD risks (9.6 vs. 8.5 %, P < 0.01) and tended to belong to the high-risk (10 year risk >10 %) group compared to those in the lowest PRAL tertile (odds ratio [OR] 1.23, 95 % confidence interval [CI] 1.22–1.35). The association between higher PRAL score and high CVD risk was stronger in the middle-aged group. Furthermore, a multiple logistic regression analysis also demonstrated this association (OR 1.20 95 % CI 1.01–1.43). Subgroup analysis stratified obesity or exercise status; individuals in unhealthy condition with lower PRAL scores had comparable ASCVD risk to people in the higher PRAL group that were in favorable physical condition. In addition, elevated PRAL scores were associated with high ASCVD risk independent of obesity, exercise, and insulin resistance, but not sarcopenia. Similar trends were observed with DAL scores.

**Conclusion:**

Diet-induced acid load was associated with increased risk of CVD, independent of obesity and insulin resistance.

## Background

Cardiovascular disease (CVD) has been a major cause of death around the world [1]. Although CVD mortality has decreased in developed countries in recent decades [1], CVD accounts for 46 % of total deaths in Europe [2] and creates high socioeconomic burdens, costing up to $320.1 billion annually in the US [3]. There is no exception for Asian countries: CVD ranks the second for mortality cause in South Korea [4], and explains 25 % for all-cause death in Japan [5]. As nutritional imbalance is a risk factor for CVD, the American Heart Association (AHA) encourages adequate intake of fruits and vegetables [3]. However, a Western diet induces an acid load that overwhelms the base production from vegetables, leading to chronic metabolic acidosis [6]. Recently, acid–base imbalance was suggested to be a risk factor for metabolic disorders [7–9]; higher dietary acid load increased the incidence of type 2 diabetes and hypertension in prospective studies [7, 8] and was associated with insulin resistance [9].

The diet-induced acid load is estimated using a formula that accounts for organic compounds, including the potential renal acid load (PRAL), dietary acid load (DAL), and net endogenous acid production (NEAP). In comparison with NEAP, which is calculated from the ratio of ingested protein and potassium, PRAL and DAL calculations include other materials (calcium, phosphorus, and magnesium) along with protein and potassium. This discrepancy accounts for the bioavailability of nutrients, enabling PRAL and DAL scores to give more accurate predictions of dietary effects on body acidity [10, 11]. Negative values of PRAL and lower DAL values indicate base-forming potential, while positive PRAL scores and higher DAL scores reflect acid-forming potential [12].

To date, studies on acid–base homeostasis have focused on the relationship between dietary patterns and the risk of hypertension or metabolic diseases, but not CVDs. We hypothesized that diet-induced acid load increases the CVD risks regardless of other metabolic conditions. The aim of this study was to investigate the association between diet-induced acid load, using both PRAL and DAL scores, with CVD risk in the general population.

## Methods

### Study population

**Fig. 1:**
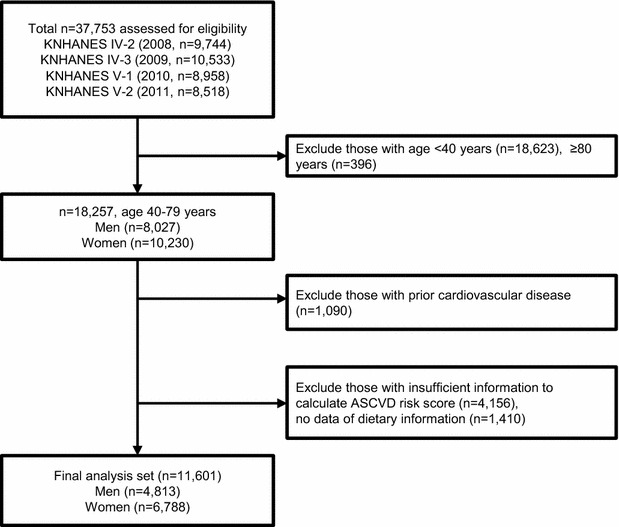
The flow diagram of subject inclusion and exclusion in the Korean National Health and Nutrition Examination Surveys (KNHANES IV and V)

This cross-sectional study extracted participant results from the Korea National Health and Nutrition Examination Surveys (KNHANES) 2008–2011. As previously described in detail, the KNHANES is a nationwide, population-based, cross-sectional health examination and annual survey. The survey is conducted by the Division of Chronic Disease Surveillance of the Korea Centers for Disease Control and Prevention in the Ministry of Health and Welfare to monitor the public health and nutrition in South Korea [13]. Each KNHANES is composed of independent data sets from the general population of Korea. As described in Fig. 1, of 37,753 participants from KNHANES 2008–2011, we initially selected those aged 40–79 without prior history of CVD. Subjects excluded were those with missing data for dietary intake and CVD risk assessment components. Ultimately, 11,601 subjects (4813 men and 6788 women) were included in the analysis. All participants provided written informed consent. The survey protocol was approved by the institutional review board of the Korean Centers for Disease Control and Prevention (2008-04EXP-01-C, 2009-07CON-03-2C, 2010-02CON-21-C, and 2011-02CON-06C).

### Measurement of clinical and laboratory parameters

KNHANES data includes a 3-part medical history, nutritional status, and laboratory tests. Subjects’ medical history was evaluated, including smoking, alcohol consumption, exercise, and disease diagnosis or treatment, based on direct interviews and self-reporting. Regular exercise was defined as more than 20 min per session and at least 3 times per week, and heavy drinkers were defined as those whose alcohol consumption was >140 g/week for men and >70 g/week for women. Blood pressure was manually measured using mercury sphygmomanometers (Baumanometer; W.A. Baum, Copiague, NY) three times on the right arms of people in resting, seated positions, and final blood pressure values were assessed by averaging the second and third blood pressure readings. Appendicular skeletal muscle (ASM) was measured using dual-energy X-ray absorptiometry (DXA, QDR 4800A; Hologic Inc., Bedford, MA, USA) and was limited in 8690 subjects. Overnight (8 h) fasting blood and spot urine samples were collected, refrigerated, and transported to the central laboratory institute (NeoDin Medical Institute, Seoul, South Korea) within 24 h. All biochemical samples were measured as previously described [14]. Estimated glomerular filtration rate (eGFR) was calculated using the Chronic Kidney Disease Epidemiology collaboration (CKD-EPI) equation [15]. The homeostasis model assessment of insulin resistance (HOMA-IR) was subtracted from the formula as previously described [16].

### Cardiovascular disease definition and assessment

CVD risk was evaluated using various approaches, including the 10 year atherosclerotic vascular disease (ASCVD) risk score from the 2013 American College of Cardiology (ACC)/AHA guidelines, as well as the Framingham 10 year CVD risk score [17, 18]. In addition, individuals exhibiting >10 % of ACC/AHA ASCVD 10 year risk and >20 % of Framingham CVD risk were classified into the high-risk group [17, 18]. Impaired fasting glucose was defined as fasting plasma glucose ranging from 100 to 125 mg/dL without prior diabetes diagnosis. Diabetes was defined in subjects who were using oral anti-diabetic agents, or exhibited fasting plasma glucose ≥126 mg/dL or glycated hemoglobin (HbA1c) ≥6.5 %. Subjects were considered to have hypertension if their systolic blood pressure was ≥140 mmHg, or their diastolic blood pressure was ≥90 mmHg, or if they were taking anti-hypertensive medications. Body mass index (BMI) was evaluated using the ratio of weight to height (kg/m^2^), and overweight was defined based on the criteria in the Asians (BMI ≥23 kg/m^2^) [19]. Chronic kidney disease (CKD) was characterized in subject with less than 60 mL/min/1.73 m^2^, accordance with CKD guideline [20]. Sarcopenia was defined as an ASM divided by BMI (ASM/BMI) <0.789 for men and <0.512 for women, as recommended by the international consensus meeting from the Foundation for the National Institutes of Health (FNIH) [21]. Because a standard definition of sarcopenia has not yet been established, ASM/height^2^ and ASM/weight estimations were applied [22, 23]. Sarcopenia was defined as <2 standard deviations (SD) below the sex-specific average for a young (age 18–39), healthy reference population from the datasets (2364 men and 3093 women) [22–24]. The ASM/height^2^ cutoff was 6.44 kg/m^2^ for men and 4.48 kg/m^2^ for women, whereas the ASM/weight cutoff was 28.80 and 22.72 % for men and women, respectively [25]. An age cutoff point to define younger or older adults was 55, the median value.

### Dietary acid load score assessment

Dietary intake information was collected by 1 day face-to-face 24 h recall method by well-trained dietary interviewers aided by various measuring devices (a common size container, standard measuring cups and spoons, a three-dimensional food model, or two-dimensional aids). Moreover, the quality control on interview at filed was conducted throughout the survey by the Center for Nutrition Policy and Promotion at the Korea Health Industry Development Institute with the checklist of the control manual. The 24 h recall method provides high-quality nutrient data and has demonstrated its accuracy in other studies [26, 27]. Detailed information and data resource profile of the survey has been provided elsewhere [28]. To calculate PRAL, intake data were needed for protein, phosphorus, potassium, calcium, and magnesium. Because magnesium intake data were not available in KNHANES, daily magnesium intake values were estimated using an external magnesium content database developed by another research group [29]. We matched the 5105 food items contained in the KNHANES 2008–2011 to the magnesium content database of 366 foods commonly consumed by Koreans. Foods not included in the database were indirectly matched with similar foods in the database. Consumption frequencies of foods by this matching process contributed 65.4 % of the total consumption frequency in KNHANES. PRAL and DAL scores were derived from the equations of nutrient intakes and tertiles of the scores were used for statistical analysis: PRAL (mEq/day) = (0.49 × protein [g/day]) + (0.037 × phosphorus [mg/day]) − (0.021 × potassium [mg/day]) − (0.026 × magnesium [mg/day]) − (0.013 × calcium [mg/day]) [30], and DAL (mEq/day) = PRAL + (body surface area [m^2^] × 41 [mEq/day]/1.73 m^2^) [10]. Both PRAL and DAL score was categorized into sex-specific tertiles. Body surface area was calculated by the Du Bois formula: 0.007184 × height^0.725^ × weight^0.425^ [31, 32]. Because data on body surface area were missing for 36 subjects, 11,565 subjects with DAL scores were newly categorized according to DAL tertiles.

### Statistical analysis

Data are presented as the mean ± SD for continuous variables, and presented as number (N), or percent (%) for categorical variables. We analyzed the study participants’ characteristics according to PRAL tertiles, using one-way analysis of variance (ANOVA) to compare continuous variables, χ^2^ tests for categorical variables followed by post hoc analyses with the Bonferroni method. To evaluate the association between acid load and CVD risk, the effects of comorbidities should be minimized. Subjects were divided according to the presence of obesity, sarcopenia, and regular exercise, and then χ^2^ tests were applied for each group. The higher PRAL group included both the second and highest PRAL tertiles, and this group was compared against the lowest PRAL tertile. Multiple logistic regression analysis was used to assess the independent association between acid load and high CVD risk (>10 % of ACC/AHA ASCVD 10 year risk score or >20 % of Framingham 10 year risk score) including other covariates. Fasting insulin, HOMA-IR, total cholesterol, triglyceride, HDL cholesterol, and LDL cholesterol values were not normally distributed; analyses were performed using log- and back-transformed data. Statistical analyses were performed using IBM SPSS version 20.0 for Windows (IBM Corp., Armonk, NY, USA); P < 0.05 was considered statistically significant.

## Results

### Diet-induced acid load and baseline characteristics

In total, 11,601 individuals free from CVD were analyzed in the present study (Fig. 1). Individuals with a higher diet-induced acid load (higher PRAL and DAL scores) tended to have increased blood pressure, triglyceride levels, and metabolic syndrome (Tables 1, 2). They also reported higher incidences of smoking cigarettes, drinking alcohol, and exercising less. Estimated dietary acid–base load also corresponded with urine acidity, reflecting the body’s acid–base homeostasis. In contrast, mean BMI, LDL, and HDL cholesterol and the proportions of CKD were similar across the PRAL tertiles. Regarding DAL scores, more discrepancies among the tertiles were observed; higher BMI, lower HDL and increased insulin resistance were found in the highest DAL tertile.

**Table 1:** Baseline characteristics of study population by categories of PRAL

	Tertiles of dietary PRAL (mEq/day)			P value^*^
	T1 (n = 3859)	T2 (n = 3540)	T3 (n = 4202)	
PRAL (mEq/d)^a^	−21.8 (−199.8 ~ −7.5)	−2.9^‡^ (−11.3 ~ 8.2)	11.2^‡^ (−10.4 ~ 181.4)	<0.001
DAL (mEq/d)	11.1 ± 20.8	35.9 ± 6.5^‡^	53.4 ± 14.6^‡^	<0.001
Magnesium (mg/d)	388.0 ± 186.4	278.1 ± 139.4^‡^	307.8 ± 177.2^‡^	<0.001
Age (year)	56.2 ± 10.3	57.1 ± 11.0^‡^	56.8 ± 11.2^‡^	0.001
Male (%)	41.4	41.3	41.8	0.881
Waist circumference (cm)	82.7 ± 9.0	82.5 ± 9.2	82.9 ± 9.3	0.255
BMI (kg/m^2^)	24.1 ± 3.0	23.9 ± 3.1^‡^	24.0 ± 3.1	0.041
Systolic blood pressure (mmHg)	120.4 ± 17.2	122.1 ± 18.0^‡^	122.2 ± 17.6^‡^	<0.001
Diastolic blood pressure (mmHg)	76.3 ± 10.1	76.8 ± 10.6	77.2 ± 10.5^‡^	0.001
Fasting plasma glucose (mg/dL)	101.4 ± 27.5	100.5 ± 23.3	100.6 ± 24.9	0.213
Total cholesterol (mg/dL)^b^	193.2 ± 36.1	194.1 ± 36.7	194.3 ± 36.5	0.436
HDL cholesterol (mg/dL)^b^	51.1 ± 12.3	51.1 ± 12.6	51.2 ± 12.5	0.842
Triglycerides (mg/dL)^b^	138.8 ± 102.7	145.5 ± 119.8^‡^	144.7 ± 113.5^‡^	0.004
LDL cholesterol (mg/dL)^b^	119.0 ± 32.4	119.2 ± 32.6	119.0 ± 32.3	0.043
Creatinine (mg/dL)	0.8 ± 0.2	0.8 ± 0.2	0.8 ± 0.3	0.903
eGFR (mL/min/1.73 m^2^)	88.3 ± 14.9	87.9 ± 15.0	88.5 ± 15.5	0.193
Insulin (ųU/mL)^b^	9.8 ± 5.2	9.8 ± 5.9	10.0 ± 6.5	0.807
HOMA-IR^b^	2.5 ± 1.9	2.5 ± 1.8	2.5 ± 2.5	0.962
Urine pH	5.8 ± 0.9	5.7 ± 0.9	5.7 ± 0.8^‡^	0.013
Heavy drink (%)	12.3	14.1	17.0^‡^	<0.001
Current smoking (%)	15.4	17.8^‡^	20.0^‡^	<0.001
Exercise (%)	28.3	24.6^‡^	24.6^‡^	<0.001
Hypertension (%)	34.4	37.9^‡^	38.5^‡^	<0.001
IFG/Diabetes (%)	23.3/13.4	24.2/13.4	25.0/12.7	0.427
Metabolic syndrome (%)	37.1	38.8	40.2^‡^	0.014
Chronic kidney disease (%)	3.8	4.3	4.5	0.263
Region (Metro/City/Rural, %)	43.3/30.2/26.5	42.3/31.5/26.3	41.7/30.2/28.1	0.237
Family history of cardio- or cerebro-vascular disease (%)	6.6	6.3	6.7	0.811

*PRAL* potential renal acid load; *DAL* dietary acid load; *BMI* body mass index; *HDL* cholesterol, high density lipoprotein cholesterol; *LDL* cholesterol, low density lipoprotein cholesterol; *eGFR* estimated glomerular filtration rate; *HOMA-IR* homeostasis model assessment of insulin resistance; *IFG* impaired fasting glucose

^‡^P < 0.05 by post hoc analyses when compared with lowest tertiles

^*^Chi square tests for qualitative variables and ANOVA tests for quantitative variables

^a^Values are medians per tertile

^b^Log-transformed

**Table 2:** Baseline characteristics of study population by categories of DAL

	Tertiles of dietary DAL (mEq/day)			P value^*^
	T1 (n = 3900)	T2 (n = 3839)	T3 (n = 3826)	
DAL (mEq/d)^a^	16.9 (−165.5 ~ 33.9)	36.1^‡^ (25.9 ~ 48.5)	51.9^‡^ (39.3 ~ 221.1)	<0.001
PRAL (mEq/d)	−27.5 ± 20.5	−2.2 ± 5.3^‡^	15.4 ± 13.4^‡^	<0.001
Magnesium (mg/d)	381.4 ± 187.5	284.0 ± 146.0^‡^	310.4 ± 177.0^‡^	<0.001
Age (year)	56.8 ± 10.5	57.4 ± 11.1^‡^	55.9 ± 10.9^‡^	<0.001
Male (%)	40.8	40.5	43.0	0.062
Waist circumference (cm)	81.7 ± 8.8	82.1 ± 9.1	84.4 ± 9.4^‡^	<0.001
BMI (kg/m^2^)	23.7 ± 2.9	23.7 ± 3.1	24.5 ± 3.2^‡^	<0.001
Systolic blood pressure (mmHg)	120.5 ± 17.3	122.2 ± 18.3^‡^	121.9 ± 17.2^‡^	<0.001
Diastolic blood pressure (mmHg)	76.1 ± 10.1	76.6 ± 10.5	77.6 ± 10.5^‡^	<0.001
Fasting plasma glucose (mg/dL)	101.3 ± 27.7	100.4 ± 24.5	100.9 ± 24.5	0.268
Total cholesterol (mg/dL)^b^	193.0 ± 36.2	194.2 ± 36.8	194.5 ± 36.2	0.146
HDL cholesterol (mg/dL)^b^	51.5 ± 12.4	51.3 ± 12.6	50.7 ± 12.3^‡^	0.031
Triglycerides (mg/dL)^b^	137.4 ± 110.9	142.8 ± 106.1^‡^	148.7 ± 118.9^‡^	<0.001
LDL cholesterol (mg/dL)^b^	118.7 ± 32.5	119.4 ± 32.8	119.0 ± 32.0	0.613
Creatinine (mg/dL)	0.8 ± 0.2	0.8 ± 0.2	0.9 ± 0.3	0.085
GFR, EPI (mL/min/1.73 m^2^)	88.3 ± 14.9	87.7 ± 15.1	88.8 ± 15.5	0.011
Insulin (μU/mL)^b^	9.5 ± 4.8	9.8 ± 5.8	10.3 ± 6.7^‡^	<0.001
HOMA-IR^b^	2.4 ± 1.8	2.5 ± 1.8	2.6 ± 2.6^‡^	<0.001
Urine pH	5.8 ± 0.9	5.7 ± 0.9	5.7 ± 0.8^‡^	0.001
Heavy drink (%)	12.1	13.9	17.8^‡^	<0.001
Current smoking (%)	15.7	17.4	20.3^‡^	<0.001
Exercise (%)	27.9	24.4^‡^	25.3	<0.001
Hypertension (%)	34.4	37.6^‡^	38.7^‡^	<0.001
IFG/diabetes (%)	22.8/13.3	24.0/13.2	25.9/13.0	0.918
Metabolic syndrome (%)	35.1	38.1^‡^	43.1^‡^	<0.001
Chronic kidney disease (%)	3.8	4.5	4.2	0.376
Region (Metro/City/rural, %)	42.5/30.2/27.3	43.2/30.6/26.3	41.6/30.9/27.5	0.613
Family history of cardio- or cerebro-vascular disease (%)	6.5	6.6	6.6	0.984

^‡^P < 0.05 by post hoc analyses when compared with lowest tertiles

^*^Chi square tests for qualitative variables and ANOVA tests for quantitative variables

*PRAL* potential renal acid load; *DAL* dietary acid load; *BMI* body mass index; *HDL* cholesterol, high density lipoprotein cholesterol; *LDL* cholesterol, low density lipoprotein cholesterol; *eGFR* estimated glomerular filtration rate; *HOMA-IR* homeostasis model assessment of insulin resistance; *IFG* impaired fasting glucose

^a^Values are medians per tertile

^b^Log-transformed

### Diet-induced acid load and cardiovascular disease risks

**Fig. 2:**
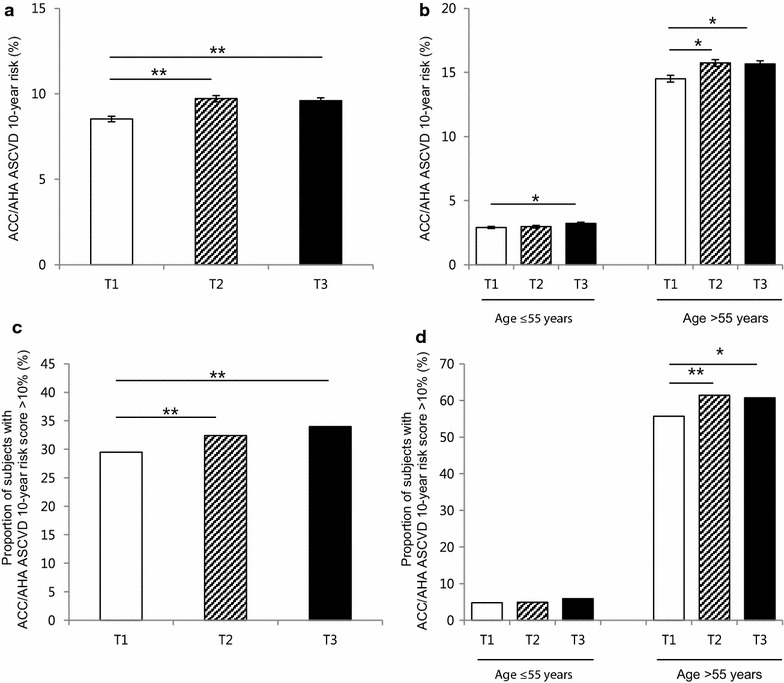
Differences in CVD risk according to PRAL tertiles. **a** Average ACC/AHA ASCVD 10 year risk scores. **b** Average ACC/AHA ASCVD 10 year risk scores in age groups. **c** Proportion of individuals with high ACC/AHA ASCVD 10 year risk (>10 %). **d** Proportion of individuals with high ACC/AHA ASCVD 10 year risk (>10 %) in age groups. Mean ± standard errors, *P < 0.05, **P < 0.001

**Fig. 3:**
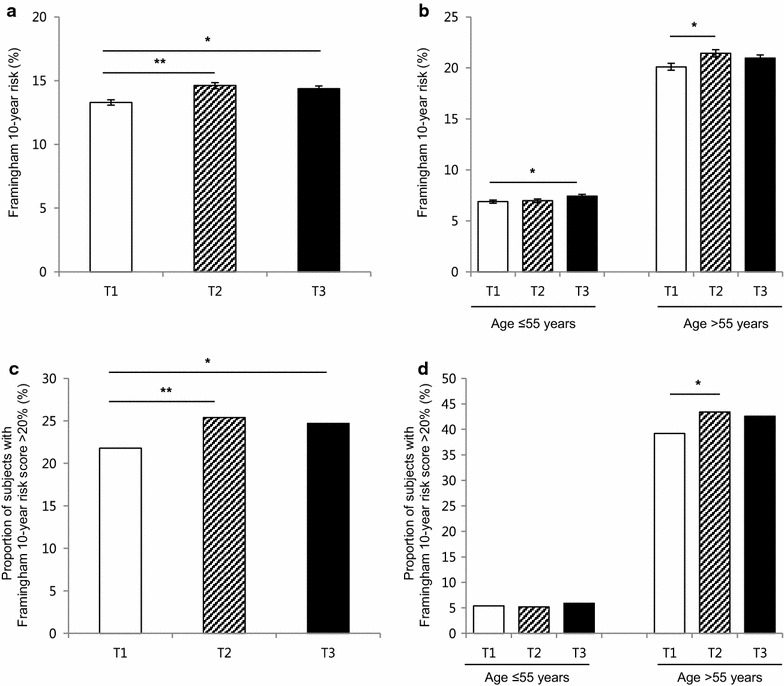
Differences in the CVD risk according to PRAL tertiles. **a** Average Framingham 10 year risk scores. **b** Average Framingham 10 year risk scores in age groups. **c** Proportion of individuals with high Framingham 10 year risk (>20 %). **d** Proportion of individuals with high Framingham 10 year risk (>20 %) in age groups. Mean ± standard errors, *P < 0.05, **P < 0.001

**Fig. 4:**
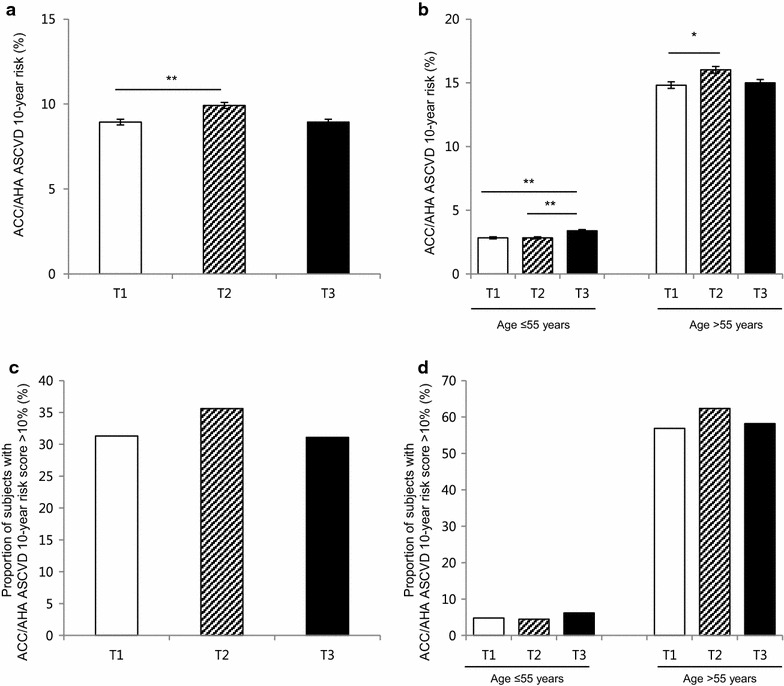
Differences in the CVD risk according to DAL tertiles. **a** Average ACC/AHA ASCVD 10 year risk scores. **b** Average ACC/AHA ASCVD 10 year risk scores in age groups. **c** Proportion of individuals with high ACC/AHA ASCVD 10 year risk (>10 %). **d** Proportion of individuals with high ACC/AHA ASCVD 10 year risk (>10 %) in age groups. Mean ± standard errors, *P < 0.05, **P < 0.001

**Fig. 5:**
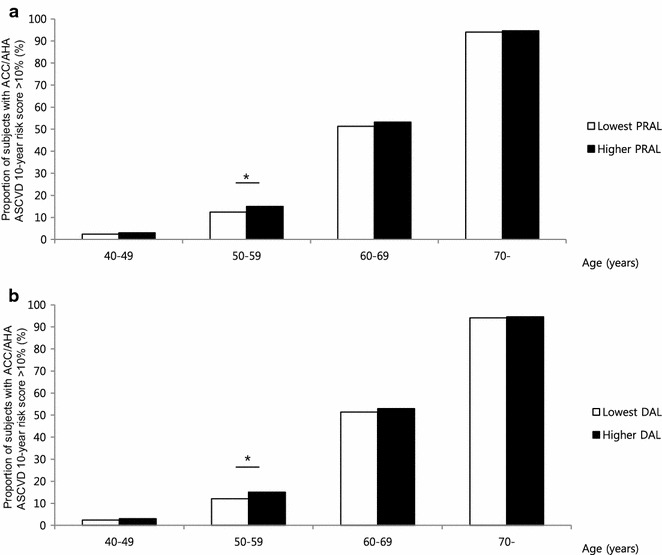
The proportion of individuals with high ACC/AHA ASCVD 10 year risk (>10 %) stratified by age groups. **a** Diet-induced acid load defined by PRAL score. **b** Diet-induced acid load defined by DAL score.* Dark* and* light boxes* indicate the high-score group and the lowest score group, respectively. *P < 0.05, **P < 0.001

The average 10 year ACC/AHA ASCVD risk score increased as PRAL increased (from 8.5 to 9.6 %, P < 0.001, Fig. 2a), and the discrepancy among the tertiles was greater in the older age group (≥55 years) (Fig. 2b). Overall, the proportion of patients in the high-risk group (predicted 10 year risk of hard ACC/AHA ASCVD event >10 %) in our population was 32.6 %, and the distribution of high-risk group members and PRAL tertiles showed the same trend as ACC/AHA ASCVD scores (29.5 and 34.0 % for lowest and highest tertiles, respectively, P < 0.001, Fig. 2c). The link between higher PRAL score and high-risk group classification was observed in older age group (Fig. 2d). Similarly, higher PRAL scores were associated with CVD risk that was estimated by a different method, the Framingham 10 year risk score (Fig. 3). Regarding DAL score, the correlation between higher DAL score and increased ACC/AHA ASCVD score was only observed in young age group (Fig. 4). As CVD risk gradually increases according to age, we stratified subjects using 10 year units, and evaluated PRAL scores and ASCVD risk scores (Fig. 5). The proportion of individuals with higher ASCVD risk was increased in the higher PRAL population, especially among middle-aged individuals (50–59 years old). A similar trend was also observed using DAL scores.

### Diet-induced acid load is linked with CVD risk independent of obesity, exercise, and insulin resistance, but not sarcopenia

**Fig. 6:**
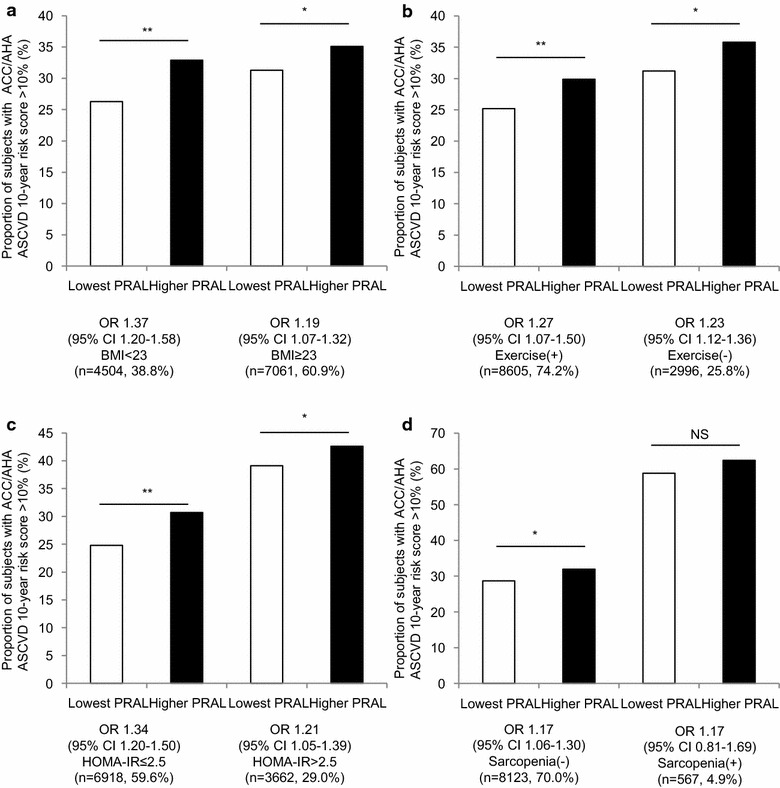
Difference in CVD risk according to PRAL scores, stratified by metabolic status and physical activity. **a** Proportion of individuals with high ACC/AHA ASCVD 10 year risk stratified by overweight defined as BMI ≥23 kg/m^2^, **b** regular exercise, **c** HOMA-IR with a cutoff point of 2.5, and **d** sarcopenia defined as ASM/BMI definition. The data are presented as OR with 95 % CI, *NS* non-significance; *P < 0.05, **P < 0.001

**Fig. 7:**
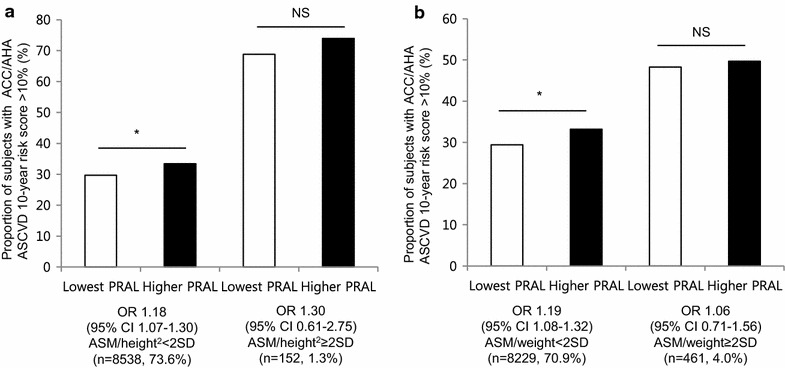
Proportion of individuals with high ACC/AHA ASCVD 10 year risk according to sarcopenic status. **a** ASM/height^2^ definition. **b** ASM/weight definition. The data are presented as OR with 95 % CI, *NS* non-significance; *P < 0.05, **P < 0.001

As overweight and low exercise are considered important CVD risk factors, we stratified the study population according to these variables. Higher PRAL scores raised the proportion of high ACC/AHA ASCVD risk regardless of BMI (Odds Ratio [OR] ranged from 1.19 to 1.37, P < 0.05 for all groups), and this association was stronger in the lean group (Fig. 6a). Interestingly, lean subjects with higher PRAL scores exhibited a similar proportion of higher CVD risk compared to those in the obese group. Although it was not statistically significant, more lean subjects with higher PRAL scores were in the ACC/AHA ASCVD high-risk group than obese subjects with lower PRAL scores. A high-PRAL diet increased ACC/AHA ASCVD risk group distribution independent of exercise status (OR ranged from 1.23 to 1.27, P < 0.05 for all groups, Fig. 6b). Moreover, the lowest PRAL individuals who did not exercise showed comparable ACC/AHA ASCVD risk with those in the higher PRAL group who regularly exercised. Increased ACC/AHA ASCVD risks in the higher PRAL group were also observed regardless of insulin resistance status assessed by HOMA-IR (Fig. 6c). Regarding skeletal muscle mass, the association between PRAL and high-risk ACC/AHA ASCVD was stronger among subjects with preserved skeletal muscle mass (OR 1.17, 95 % CI 1.06–1.30, Fig. 6d); this association was not observed among sarcopenic subjects (OR 1.17, 95 % CI 0.81–1.69). This trend was consistently observed when other sarcopenia definitions were applied (Fig. 7).

### Higher PRAL and DAL scores are associated with elevated predicted risk of CVD regardless of hypertension or diabetes

We assessed high predicted ASCVD risk and diet-induced acid load after adjusting for confounding factors, including age, sex, and other clinic-laboratory parameters. Multiple logistic regression analysis showed that higher PRAL scores was independently associated with high risk of CVD evaluated from both ACC/AHA assessment and Framingham risk score (Tables 3, 4). The effects of PRAL on ACC/AHA ASCVD risk showed no significant difference in patients categorized by hypertension, by age or by overweight (hypertension; P = 0.345, age; P = 0.112, overweight; P = 0.150, respectively, for interaction). Although this statistical significance was weakened in DAL score, the similar trend was observed in DAL tertiles (Table 5).

**Table 3:** Odds ratio and 95 % confidential interval of high ASCVD risk (>10 % 2013 ACC/AHA score) according to PRAL tertiles in adults

	Lowest tertile	Second tertile	Highest tertile
Crude	1 (referent)	1.27 (1.15–1.40)	1.23 (1.22–1.35)
Model 1	1 (referent)	1.29 (1.11–1.51)	1.18 (1.02–1.36)
Model 2	1 (referent)	1.28 (1.10–1.50)	1.17 (1.01–1.35)
Model 3	1 (referent)	1.31 (1.09–1.58)	1.20 (1.01–1.43)

Model 1: adjusted for age (per 5 years), and sex

Model 2: adjusted for age (per 5 years), sex, exercise, and family history of cardio- and cerebro-vascular disease

Model 3: adjusted for age (per 5 years), sex, exercise, family history of cardio- and cerebro-vascular disease, diabetes, hypertension, LDL cholesterol, eGFR, and urine pH

*ASCVD* 10 year atherosclerotic vascular disease; *PRAL* potential renal acid load; *eGFR* estimated glomerular filtration rate; *LDL* cholesterol; low density lipoprotein cholesterol

**Table 4:** Odds ratio and 95 % confidential interval of high ASCVD risk (>10 % 2013 ACC/AHA score) according to DAL tertiles in adults

	Lowest tertile	Second tertile	Highest tertile
Crude	1 (referent)	1.21 (1.10–1.33)	1.00 (0.90–1.09)
Model 1	1 (referent)	1.24 (1.07–1.44)	1.16 (1.00–1.35)
Model 2	1 (referent)	1.23 (1.06–1.43)	1.15 (0.99–1.34)
Model 3	1 (referent)	1.25 (1.04–1.46)	1.07 (0.89–1.28)

Model 1: adjusted for age (per 5 years), and sex

Model 2: adjusted for age (per 5 years), sex, exercise, and family history of cardio- and cerebro-vascular disease

Model 3: adjusted for age (per 5 years), sex, exercise, family history of cardio- and cerebro-vascular disease, diabetes, hypertension, LDL cholesterol, eGFR, and urine pH

*ASCVD* 10 year atherosclerotic vascular disease; *DAL* dietary acid load; *eGFR* estimated glomerular filtration rate; *LDL* cholesterol; low density lipoprotein cholesterol

**Table 5:** Odds ratio and 95 % confidential interval of high Framingham 10 year risk (>20 %) according to PRAL tertiles in adults

	Lowest tertile	Second tertile	Highest tertile
Crude	1 (referent)	1.22 (1.10–1.36)	1.18 (1.06–1.31)
Model 1	1 (referent)	1.19 (1.03–1.36)	1.16 (1.01–1.32)
Model 2	1 (referent)	1.18 (1.03–1.36)	1.15 (1.01–1.31)
Model 3	1 (referent)	1.25 (1.05–1.49)	1.19 (1.01–1.41)

Model 1: adjusted for age (per 5 years), and sex

Model 2: adjusted for age (per 5 years), sex, exercise, and family history of cardio- and cerebro-vascular disease

Model 3: adjusted for age (per 5 years), sex, exercise, family history of cardio- and cerebro-vascular disease, diabetes, hypertension, LDL cholesterol, eGFR, and urine pH

*ASCVD* 10 year atherosclerotic vascular disease; *PRAL* potential renal acid load; *eGFR* estimated glomerular filtration rate; *LDL* cholesterol; low density lipoprotein cholesterol

## Discussion

In this current large, nationally representative, population-based study, we demonstrated that people with higher diet-induced acid load had higher CVD risks in the general population. Diet-induced acid load was closely linked with CVD risk especially among middle-aged individuals. In addition, higher PRAL scores were associated with CVD risk independent of obesity, exercise, and insulin resistance, but not sarcopenia. This association remained significant after adjusting for other essential confounding factors.

To date, the health effects of acid–base imbalance were mainly investigated in bone mass [33], and kidney stones [34]. Recently, studies on systemic metabolism were conducted that extended the effects of diet-induced acid load on one’s body. For example, a prospective study showed that higher PRAL scores correlated with the incidence of type 2 diabetes (hazard ratio = 1.56, 95 % CI 1.29‒1.90) [7]. Similarly, a Japanese study reported men with highest PRAL quartile had 61 % increased type 2 diabetes prevalence in over 5 years follow-up period [35]. In addition, individuals with higher diet-induced acid load were reported to exhibit 27 % increases on hypertension development regardless of age [8], and this positively correlated with the prevalence of nonalcoholic fatty liver disease [36]. In this study, we also demonstrated higher incidences of increased blood pressure, prevalence of hypertension and metabolic syndrome in conjunction with higher PRAL and DAL scores. In addition, individuals with higher diet-induced acid load tended to have unhealthy lifestyle patterns during relatively younger ages, including less physical activity, stricter adherence to Western diet patterns, and higher BMI, in accordance with previous studies [7, 8, 36]. This was consistent with our findings that in the highest PRAL score group, individuals were more likely to exhibit sedentary patterns, increased waist circumferences, and high prevalence of smoking and drinking alcohol.

### Diet-induced acid load, cardiovascular diseases and possible mechanisms

The mechanism linking diet-induced acid load and metabolic disease incidence is mainly reported as insulin resistance [7, 9]. Toward that end, insulin binding affinity to its receptor was markedly decreased in individuals with metabolic acidosis [37]. Thus, even in healthy individuals, a very low degree of metabolic acidosis could lead to insulin resistance, resulting hyperglycemia [38]. Moreover, a previous study reported that attenuating metabolic acidosis could increase insulin sensitivity [39]. In accordance with this evidence, our study results demonstrated that the highest DAL tertile was associated with increased values of HOMA-IR. Regarding the effect of diet-induced acid load on blood pressure, several mechanisms have been suggested. A diet depleted of potassium could affect vasodilatation and be toxic to the blood vessels [40]. Potassium restriction results in intracellular potassium deficits and causes compensatory sodium gains in cells to maintain tonicity and volume [40]. In a human study, even a 10 day period of low potassium intake increased systolic blood pressure by 5 mmHg (P < 0.02) and modified salt sensitivity, resulting in exacerbation of hypertension [41]. Conversely, hypertensive rats fed a potassium-rich diet showed decreases in blood pressure and stroke development [42, 43]. Likewise, our study demonstrated that PRAL and DAL scores were positively associated with both systolic and diastolic blood pressure values, as well as hypertension prevalence (OR 1.19, 95 % CI 1.09‒1.31). In addition, systemic metabolic acidosis, caused by the Western diet, is associated with excessive cortisol levels and leads to ammoniagenesis, which may lead to loss of renal function [12, 44]. Diet-induced insulin resistance could autonomously promote cardiovascular disease in various pathways; impair coronary microcirculatory function [45], stimulate conduction dysfunction and increase arrhythmogenesis [46].

### Diet-induced acid load in unhealthy states

Previously, the correlation between diet-acid load and metabolic disease was stronger in non-obese individuals, suggesting that it is independent of adiposity [7, 9]. We evaluated the PRAL score and ACC/AHA ASCVD risk according to BMI as well as insulin resistance. Although this association remained significant in both lean/overweight and insulin sensitive/resistant groups, more substantial correlations were observed in lean individuals (OR 1.37 vs 1.19) and in the insulin sensitive group (OR 1.34 vs 1.21). Moreover, there was no significant difference in risk of ACC/AHA ASCVD between lean subjects with higher PRAL scores and those in the overweight group in the lowest PRAL tertile, suggesting that CVD risk of lean individuals would increase if diet-induced acid loads were elevated. An epidemiologic study that reported that dietary patterns could increase the risk of obesity, even in lean individuals, suggests that diet affects metabolism in non-obese populations [47]. In addition, if sedentary individuals lowered their intake of dietary acid, they might exhibit comparable CVD risks with people who regularly exercised. While obesity and exercise are major contributing factors to CVD risk, diet-induced acid load might modify these effects. Regardless, the association between diet-induced acid load and CVD risk with respect to muscle mass seems compelling. There has been some evidence that sarcopenia could influence CVD, such as increased arterial stiffness and inflammatory markers in people with sarcopenia [48, 49]. Our results also demonstrated increased CVD risk in the sarcopenic group compared to the non-sarcopenic group; however, the association between PRAL score and ACC/AHA ASCVD risk did not remain significant among sarcopenic individuals. Furthermore, subjects with higher PRAL scores but preserved muscle mass had lower CVD risk than those in the sarcopenic group with lower PRAL scores. This might suggest that skeletal muscle mass is more closely associated with lower CVD risks compared to healthy diet patterns.

This study has some limitations. First, our cross-sectional study design could not conclude causality between diet-induced acid load and CVD risk. Second, PRAL and DAL scores were derived from self-reports using a 24 h recall method, which only confirmed the short term dietary intakes of the study participants; the daily variation in food consumption could not be considered. Third, we could not assess the quality of protein that was used to calculate PRAL and DAL scores. In addition, KHNASE did not collect each individual’s medication information, and not evaluate physical performance status, and muscle strength. Sarcopenic status was classified by muscle mass alone, not considering muscle strength or physical performance. The potential medication effect on CVD risk was not considered in our results.

Despite these limitations, the current study had several strengths. First, it was a large population study based on national data, guaranteeing the statistical reliability of results. As KNHANES represents the non-institutionalized general population, selection bias was minimized. Second, this investigation provided strong evidence of a close relationship between diet-induced acid load and CVD risk by adjusting for other confounding factors and conducting stratification analyses. To the best of our knowledge, this is the first large-scale study to estimate the CVD risk associated with diet-induced acid load. We assessed diet-induced acid load using variable methods: PRAL and DAL scores. The correlation between PRAL and DAL scores was high (Pearson correlation coefficient = 0.99, P < 0.001) in our study, which was consistent with previous studies [10]. Third, individual CVD risks were estimated using various equations, and comparable results were derived regardless of these different models. Fourth, although the current study was cross-sectional, we limited the study population by excluding those with prior CVD history to prevent reverse causality.

In the light of dietary pattern and CVD risk, our study results demonstrate that individuals with higher diet-induced acid load are more vulnerable to CVD risks. Despite advances in CVD diagnostic and treatment modalities, prevention is the most high-leverage action, and therefore, modifiable risk factors are important. Prospective, well-designed, longitudinal studies with sufficient laboratory and cardiovascular-imaging resources are warranted to elucidate the complex relationship between diet-induced acid load and CVD risk. If the causality between diet-induced acid load and CVD risk, as well as the cutoff value of diet-induced acid load are clearly investigated, then meaningful, comprehensive, and practical dietary recommendations would be provided to the public.

## Conclusions

This nationwide survey of a representative sample of the Korean population demonstrated that diet-induced acid load was associated with increased risks of CVD, independent of other cardiovascular factors.

## Abbreviations

ACC/AHA: American College of Cardiology/American Heart Association
ASCVD: atherosclerotic vascular disease
BMI: body mass index
CI: confidential interval
CVD: cardiovascular disease
DAL: dietary acid load
HDL: high density lipoprotein
HOMA-IR: homeostasis model assessment of insulin resistance
KNHANES: The Korea National Health and Nutrition Examination Surveys
LDL: low density lipoprotein
PRAL: potential renal acid load
